# Neuropsychological and quality of life outcomes in PKU patients: expert recommendations of assessment tools in Brazil

**DOI:** 10.1055/s-0043-1768677

**Published:** 2023-06-19

**Authors:** Ida Vanessa Doederlein Schwartz, Andrea Amaro Quesada, Erlane Marques Ribeiro, Ana Maria Martins, Daniel Reda Fenga Vilela, André Pessoa

**Affiliations:** 1 Universidade Federal do Rio Grande do Sul, Porto Alegre RS, Brazil.; 2 Hospital de Clínicas de Porto Alegre, Porto Alegre RS, Brazil.; 3 Universidade de Fortaleza, Fortaleza CE, Brazil.; 4 Hospital Albert Sabin, Fortaleza CE, Brazil.; 5 Universidade Federal de São Paulo, São Paulo SP, Brazil.; 6 BioMarin Brasil Farmacêutica Ltda., São Paulo SP, Brazil.; 7 Universidade Estadual do Ceará, Fortaleza CE, Brazil.

**Keywords:** Phenylketonurias, Mental Health, Neuropsychological Tests, Quality of Life, Brazil, Fenilcetonúrias, Saúde Mental, Testes Neuropsicológicos, Qualidade de Vida, Brasil

## Abstract

**Background** Phenylketonuria (PKU) is an inborn error of metabolism caused by deficient activity of phenylalanine hydroxylase. In Brazil, the National Neonatal Screening Program enables early treatment of patients with PKU, which prevents them from developing severe neurological damage and mental disabilities. However, between 20 and 30% of early-treated patients with PKU present focal cognitive deficits, including deficits in working memory, processing speed, and psychiatric symptoms such as anxiety, depression, and attention deficit hyperactivity disorder (ADHD). Therefore, age-specific neuropsychiatric and cognitive tests are important components of PKU patient care. To date, there are no officially approved guidelines or recommendations of tools in Portuguese validated for use in Brazil that could be applied to assess these parameters in patients with PKU.

**Objective** To recommend tools validated for use in Brazil that can be used in daily clinical practice to assess quality of life and neuropsychological outcomes in patients with PKU.

**Methods** Six Brazilian experts discussed about eligible tools based on their clinical experience, the feasibility of their use in clinical routines, and their availability in public health services. Before the meeting, an independent review of the literature was conducted to identify the currently validated tools in Brazil, using the MEDLINE and SciELO databases.

**Results** The experts recommended nine tools to assess quality of life (Peds-QL, SF-36 or WHOQOL-bref), executive function (BRIEF or Bayley-III), IQ (SONR 2½-7[a] or WASI) and ADHD (MTA-SNAP-IV and ASRS).

**Conclusion** These instruments may be easily incorporated into clinical practice and improve the quality of multidisciplinary care of patients with PKU.

## Resumo

**Antecedentes** A fenilcetonúria (PKU) é um erro inato do metabolismo causado pela atividade deficiente da fenilalanina hidroxilase. No Brasil, o Programa Nacional de Triagem Neonatal permite o tratamento precoce de pacientes com PKU, o que os impede de desenvolver danos neurológicos e deficiências intelectuais graves. No entanto, já foi descrito que de 20 a 30% dos pacientes tratados precocemente com PKU apresentam déficits cognitivos focais, incluindo déficits na memória de trabalho, velocidade de processamento e sintomas psiquiátricos como ansiedade, depressão e Transtorno de Déficit de Atenção e Hiperatividade (TDAH). Neste sentido, testes neuropsiquiátricos e cognitivos são componentes importantes no cuidado destes pacientes. Atualmente, não existe um compêndio científico ou recomendações de ferramentas validadas em português para avaliar a saúde mental em pacientes brasileiros com PKU.

**Objetivo** Recomendar ferramentas validadas localmente para avaliar a qualidade de vida e aspectos neuropsicológicos de pacientes com PKU.

**Métodos** Seis especialistas brasileiros discutiram as ferramentas mais apropriadas com base em suas experiências clínicas, a viabilidade de realizar as avaliações na rotina clínica, e o acesso às ferramentas na saúde pública. Antes da reunião, foi realizada uma revisão independente da literatura para identificar as ferramentas validadas no Brasil, utilizando as bases de dados MEDLINE e Scielo.

**Resultados** Os especialistas recomendaram nove ferramentas para avaliar a qualidade de vida (Peds-QL, SF-36 ou WHOQOL-bref), função executiva (BRIEF ou Bayley-III), QI (SONR 2½-7[a] ou WASI) e TDAH (MTA-SNAP-IV e ASRS).

**Conclusão** Estes instrumentos podem ser facilmente incorporados na prática clínica e melhorar a qualidade dos cuidados multidisciplinares dos pacientes com PKU.

## INTRODUCTION

Phenylketonuria (PKU) is an autosomal recessive inborn error of metabolism (OMIM # 261600) caused by pathogenic sequence variants in the *PAH* gene that lead to deficient activity of phenylalanine hydroxylase (PAH), which increases phenylalanine (Phe) levels in the blood. 1 The incidence of PKU varies worldwide, ranging from 1:10,000 to 1:125,000. 2 In South America, the prevalence of PKU varies from ∼ 1:25,000 to 50,000 live births. 3 The most common signs and symptoms in untreated patients with PKU include intellectual disability, seizures, behavioral disturbance, psychiatric symptoms, motor disturbances, pyramidal signs, microcephaly, eczematous rash, and movement disorders such as ataxia and Parkinsonism. 4

High blood Phe levels can easily cross the blood-brain barrier, leading to damage to brain development and function, such as cognitive deficits and memory impairment. More specifically, increased Phe levels compromise the transport of tyrosine (Tyr) and tryptophan (Trp) to the brain by LAT1, creating a hypomonoaminergic state. 5 In addition, PKU is thought to be associated with suboptimal dopamine and noradrenaline levels, and reduced serotonin levels; it is possible that the competition for transporter capacity at the blood–brain-barrier may also affect Trp levels, which leads to the lower serotonin levels. Disorders in serotonergic neurotransmission are strongly associated with mood disorders and mental health problems. 6 Therefore, as a consequence of these metabolic disturbances, intellectual disability, executive function impairments, attention deficit hyperactivity disorder (ADHD), and behavioral and psychiatric disorders such as depression and anxiety may develop in PKU patients. Collectively, the negative impact on mental health can potentially lead to a poor quality of life in patients with PKU. 4 7 8 9 10

To reduce Phe levels and prevent the progression of clinical alterations, a lifelong treatment of PKU is recommended. 11 12 Since Phe is mainly obtained through diet, available treatments require dietary restriction – the low-Phe diet for PKU patients is often combined with Phe-free L-amino acid supplements. Pharmacological treatments to assist in controlling blood Phe levels are also available, including the use of sapropterin dihydrochloride (Kuvan, BioMarin Pharmaceutical Inc., Novato, CA, USA), a synthetic analogue of tetrahydrobiopterin (BH _4_ ) 13 14 and pegvaliase (Palynziq, BioMarin Pharmaceutical Inc., Novato, CA, USA) for adult patients. 15 16

In Brazil, the National Neonatal Screening Program enables early dietary treatment of patients with PKU, which prevents them from developing severe neurological damage and mental disabilities. However, 20 to 30% of early-treated patients with PKU present focal cognitive deficits, 17 18 including deficits in working memory, processing speed and psychiatric symptoms such as anxiety, depression, and ADHD. 17 18 19 20 21 22 23 24 25 26 A strong correlation between Phe levels and processing speed has been observed in children with PKU, but not in adults. 27 Many factors contribute to increasing the risk of developing neurocognitive or psychological symptoms in PKU patients, such as *PAH* variants, onset of therapy, lifelong Phe levels and adherence to treatment. 16 28

Besides appropriate intellectual and mental health assessments, age-specific neuropsychiatric and cognitive testing are important components of care for individuals affected with PKU. The American College of Medical Genetics and Genomics (ACMG) guidelines provide a list of recommended tests to assess developmental and intellectual parameters, executive functioning, and behavioral/emotional and adaptive skills according to the patient's age, and at the indicated interval of testing. 11 In the European guidelines for the diagnosis and management of PKU, it is recommended that routine neurocognitive assessments should be performed at the ages of 12 years old and 18 years old in all patients. During clinic visits, quality of life and psychosocial functioning should be assessed through PKU-specific health-related quality of life (PKU-QoL) instruments. Examination of adaptive skills and a psychiatric evaluation are also recommended. 12

The Brazilian Ministry of Health recently published the Brazilian Phenylketonuria Clinical Protocol and Therapeutic Guidelines (Protocolo Clínico e Diretrizes Terapêuticas da Fenilcetonúria, 2020), recommending a pool of medical procedures and therapies for PKU patients in the country. The protocol requires, among other parameters, the monitoring of the quality of life and neuropsychological functions of PKU patients, but with no recommendations about the appropriate assessment tools the clinicians should use for the effective follow-up of this population in Brazil. The evaluation of neurocognitive and neuropsychological parameters in PKU patients is not routinely performed, mainly due to the lack of standardized clinical guidelines and a scarcity of neuropsychologists at nonspecialized centers. Moreover, to date, there are no officially approved guidelines or recommendations in the medical literature regarding neuropsychological tools in Portuguese validated for use in Brazil that could be applied to evaluate patients with PKU.

Therefore, to improve the quality of care of PKU patients and contribute to adherence to the protocol set out by the Ministry of Health and the local government protocol, the aim of the present article was to describe and recommend locally validated tools for neuropsychological assessment of patients with PKU in daily clinical practice.

## METHODS

A group of six Brazilian experts in PKU met virtually on May 21 ^st^ , 2021, to discuss the most appropriate validated tools for the neuropsychological assessment of patients with PKU. The group consisted of three geneticists, one pediatric neurologist, one neuropsychologist, and one medical and scientific moderator (a pediatrician and a PKU specialist). The experts were selected based on their knowledge of the subject matter and their experience in managing PKU patients in Brazil. At this meeting, the discussion was focused on selecting the available tools (scales and questionnaires) currently validated in Brazil, based on three criteria: (i) the experts' clinical experience with the tools in their daily practice – the selected tools should have already been used by the experts, (ii) the feasibility of performing the assessments as part of the clinical routine – the tools should not require very specific conditions, and (iii) and the access to the tools – they should be easily available for use by public health professionals.

Before the meeting, an independent review of the literature was conducted to identify the available tools (scales and questionnaires) currently validated in Brazil, following the aspects required by the Brazilian protocol about quality of life and the neuropsychological functions of PKU patients. The following search keywords (in Brazilian Portuguese) were used to find locally validated tools: *quality of life assessment, health-related quality of life, neuropsychological assessment, intellectual assessment, phenylketonuria* . The MEDLINE and SciELO databases were searched, with an unlimited search period.

The authors discussed the various tools through an online platform (Within3) and came to an agreement on which should be recommended for use in Brazil. An external moderator and a medical writer contributed to the production of text that reports the results of the positions of the authors.

## RESULTS

The recommendations of the experts are summarized in Table 1 and comprise nine tools that can be useful to assess quality of life and neuropsychological outcomes in patients with PKU in daily clinical practice. Recommended assessments are classified by domains and patient age.

**Table 1:** Recommendations of validated tools for quality of life and neuropsychological assessment of patients with PKU in Brazil

Evaluation domain *According to local protocol of PKU management*	Recommended tool	Period assessed by the questionnaire	Appropriate ages and administration	HCP/HRP who can apply the tool
**Quality of life**	PedsQL	Last month	Children and adolescents *between 5 and 18 years old* Parent questionnaires for children and adolescents *between 2 and 18 years old*	General practitioner, Pediatrician
	SF-36	Last month	It can be self-administered by persons ≥ *14 years old* , or administered by a trained interviewer in person or by telephone	General Practice
	WHOQOL-bref	Last 2 weeks	*For individuals > 12 years old in Brazil* It can be self-administered if respondents have sufficient ability; otherwise, it should be interviewer-assisted or interview-administered	General practitioner, Pediatrician
**Cognition & motor function**	Bayley-III	Current	Administered by a trained professional for *children up to 42 months old*	Neurologist, Neuropsychologist
**Neuropsychological function**	BRIEF (Executive functioning)	Last 6 months	Parent questionnaires for children and adolescents *between 5 and 18 years old*	Neurologist, Neuropsychologist
	WASI	Current	Tests for persons from *6 to 89 years old*	Neuropsychologist
	SONR 2½-7[a]	Current	Administered by a trained professional, for c *hildren aged 2.5 to 7 years old*	Neuropsychologist
	MTA-SNAP-IV (ADHD)	Last 12 months	Administered by parents or teachers from 5 to 17 years old children and adolescents	Neurologist, Neuropsychologist
	ASRS (ADHD)	Last 12 months	Self-administered, for persons > 17 years old	Neurologist, Neuropsychologist

Abbreviations: ADHD, Attention deficit hyperactivity disorder; HCP, health care professionals (physicians); HRP, health-related professionals (psychologists, occupational therapeutics).

### Assessment of quality of life

Despite the significantly improved outcomes regarding early and continuously treated PKU patients, they still need to cope with the psychological and social burden of having a chronic disorder, as well as the impact of the restrictive dietary treatment. Together, these factors can affect the quality of life of patients and their families. 11

A specific tool to evaluate the quality of life of patients with PKU and their caregivers, the PKU-QOL questionnaire, has already been developed and validated in eight countries. It comprises four PKU-QOL questionnaires developed for children, adolescents, and adults with PKU, and for the parents of children with PKU. It assesses three domains: PKU symptoms, PKU in general (i.e., the physical, emotional, social, and overall burden of PKU), and the impact of treatment. 29 30

In the European guidelines for the diagnosis and management of PKU, it is the only tool specifically recommended for patient assessments. 12 Through the use of the PKU-QOL questionnaire, it has already been shown that PKU has negative impacts on patients' lives, mainly related to the emotional impact of the disease and its management (some patients feel anxious about controlling their blood Phe levels or guilt about poor adherence to dietary treatment). 31

In Brazil, the PKU-QOL tool has already been translated and linguistically validated 32 ; however, the validation of the questionnaire with Brazilian patients has not yet been performed.

Although disease specific health-related quality of life (HRQoL) assessments are undoubtedly more accurate to evaluate small changes and the impact of different interventions or treatments, general approaches are useful to explore, describe, and compare populational characteristics. Therefore, in the following section, we present the three assessment tools recommended by the authors. Although they are not disease specific, they can be used to evaluate the quality of life in patients with PKU.

#### Peds-QL

The Pediatric Quality of Life Inventory (Peds-QL) is a modular approach to evaluating pediatric HRQoL, with generic modules and modules focused on specific diseases. 33 Physical health, emotional functioning, social functioning, and school functioning (four domains) are evaluated through a *Likert* scale questionnaire, and the emotional, social, and school dimensions are evaluated through the psychosocial health summary score. All items are reverse-scored and linearly transformed to a 0–100 scale, with higher scores representing better quality of life. 34

The PedsQL 4.0 generic questionnaire that includes a self-assessment for children and adolescents between 5 and 18 years old and parent questionnaires for children and adolescents between 2 and 18 years old. 34 For patients between 5 and 8 years old, it can be completed by interviewing the patient, and for patients between 2 and 5 years old, parents or guardians should complete the questionnaire. 35

The PedsQL 4.0 generic questionnaire was translated into a Brazilian Portuguese version and was validated for use with the Brazilian population in 2008, 36 allowing its application to a variety of patients, including those with PKU. 37 It was originally proposed to be self-administered, 33 but the validation study for the Brazilian population showed that it should be administered by the interviewer. 36 The application of the instrument and the calculation of total scores and scales were considered to be fast and easy. 38

As a generic questionnaire, PedsQL is not as sensitive as specific HRQoL instruments; nevertheless, it may generate important insights into the characteristics of the population being evaluated. A multicenter study of PKU European patients found no significant difference in HRQoL scores when compared with reference values from the US population; the study included children, adolescents, and adults with PKU. 31 However, a Brazilian study with PKU patients found lower overall HRQoL scores in the PedsQL than in their respective controls. This discrepancy may be due to possible differences in the cognitive performance of both populations since the Brazilian study included patients with cognitive disabilities, despite the early initiation of treatment with a suboptimal adherence to treatment. 12 37

More information about PedsQL, including about copyrights and full versions of the instrument may be obtained from the PedsQL website - Measurement Model for the Pediatric Quality of Life Inventory. 39 Versions of the instrument and permission for use may be requested from the Mapi Research Trust. 40

#### SF-36

The Medical Outcomes Short-Form Health Survey (SF-36) was designed in English for use in clinical practice and research, health policy evaluations, and general population surveys. The SF-36 assesses 8 health concepts, scored as 8 multi-item scales: 1) limitations in physical activities because of health problems; 2) limitations in social activities because of physical or emotional problems; 3) limitations in usual role activities because of physical health problems; 4) bodily pain; 5) general mental health (psychological distress and well-being); 6) limitations in usual role activities because of emotional problems; 7) vitality (energy and fatigue); and 8) general health perceptions. 41

The questionnaire presents a final score from 0 to 100, where 0 corresponds to the worst general health condition and 100 to the best condition. It can be self-administered by persons 14 years old and older, or administered by a trained interviewer in person or by telephone. 41

A Brazilian Portuguese version was developed and cross-culturally adapted, and its measurement properties of reliability and validity were first evaluated in Brazilian patients with rheumatoid arthritis. 42 A large study with 12,423 randomly selected Brazilian men and women aged ≥ 18 years old showed that SF-36 scores vary according to age and gender and provide normative data for the Brazilian population. 43 These data are essential to compare the health status of the general population and of patient populations, as well as the effect of interventions on HRQoL. The Brazilian version of the SF-36 may be obtained online. 44

The SF-36 has been used along with other tools to evaluate quality of life in PKU patients and parents in Tunisia 45 and the United Kingdom (UK). 46 In the Tunisian study, their findings on altered quality of life were used to develop a psychological and social support strategy for at-risk parents, which could also be an interesting approach in Brazil.

#### WHOQOL-BREF

The WHOQOL-BREF is a shortened version of the WHOQOL-100 developed by the WHO (World Health Organization) Quality of Life Group. It consists of 26 items that evaluate four domains: physical health, psychological health, social relationships, and environment. It also includes one facet on overall quality of life and general health. 47 According to the authors, the WHOQOL-BREF will be most useful in studies that require a brief assessment of quality of life and may be of use to health professionals in the assessment and evaluation of treatment efficacy – which could bring important insights regarding the PKU patient context.

If the patient has sufficient ability, the WHOQOL-BREF should be self-administered; otherwise, a trained interviewer must administer the specific forms. When the assessment is interviewer-administered, standard instructions should be read out to the respondents. 48

The WHOQOL-BREF has been validated in Brazil in a heterogeneous sample of patients > 12 years old with different diseases and treated in both outpatient and inpatient settings. The psychometric characteristics of the WHOQOOL-BREF Portuguese version were similar to those of the multicenter study used to develop the instrument. 47 49

Regarding the assessment of PKU patients and their caregivers, the WHOQOOL-BREF has already been used in several studies in different populations. 50 51 52 53 When compared with the general population, all of them found that PKU patients and caregivers present lower overall quality of life. In addition, important factors that have been correlated with lower quality of life are high levels of depression and anxiety, and the caregiver's occupation (especially unemployment). 51 Results from PKU patients and caregivers emphasize the need to follow-up quality of life parameters and helps to identify different opportunities to the management and treatment of the disease.

### Neuropsychological assessment

Many neuropsychological tools to assess mental health are currently available, but not all of them have been validated for use in Brazil. The ACMG Guideline recommendations focus on developmental and intellectual, executive function, behavioral and emotional, and adaptive skills evaluations. 11 On the other hand, the European guidelines recommend the evaluation of: IQ, perception/visuospatial functioning, EF (divided into inhibitory control, working memory and cognitive flexibility) and motor control. 12

Considering these international guidelines and the availability of validated tools in Brazil, the authors recommended the following tools to be used according to the patient's age: Bayley-III (Bayley Scales of Infant Development III), BRIEF (Behavior Rating Inventory of Executive Function), WASI (the Wechsler Abbreviated Scale of Intelligence) and SON-R 2½-7[a].

#### Bayley scales of infant development III (Bayley-III)

The Bayley-III is the gold standard for the assessment of the development of infants aged 1 to 42 months old. It comprises five scales: cognitive, language, motor, social-emotional, and adaptive behavior. However, only cognitive, language and motor parameters are assessed through direct observation of the child; the other domains (social-emotional and adaptive behavior) are assessed through questionnaires completed by the main caregiver. Bayley-III should be administered by a trained professional; the average time taken to complete varies with age and ranges from ∼ 50 to 90 minutes. The assessment provides raw and scaled scores for each domain, as well as composite scores and percentile ranks for each scale. 54

In 2016, the Bayley-III was translated into Brazilian Portuguese and culturally adapted for use with Brazilian children. 55 To our knowledge, there have as yet been no studies using the Bayley-III to evaluate Brazilian PKU patients.

In the international literature, it has been shown that PKU patients treated with sapropterin dihydrochloride (after determination of responsiveness) maintained their baseline Bayley-III scores, indicating that no child appeared to be at risk for developmental delay during the study. The test had been administered every 6 months to children aged 0 to < 30 months old. 56 In addition, a study with early-treated Chinese PKU patients showed they had no delay in mental and motor development when compared with a healthy control group, assessed by Bayley-III. 57 Moreover, the test has also been used to evaluate the effect of maternal PKU on the neurocognitive development of the offspring. 58

#### Behavior rating inventory of executive function (BRIEF)

The Behavior Rating Inventory of Executive Function (BRIEF) is a standardized instrument that allows the observer to rate the executive function of 5- to 18-year-old children and adolescents – it is a questionnaire for parents, teachers and adolescents. It consists of eight clinical scales assessing dimensions of executive function entitled: inhibit, shift, emotional control, initiate, working memory, plan/organize, task monitor, and organization of materials. 59

The form for parents and teachers consists of 86 questions each, which should be answered by parents and teachers of children and adolescents between 5 and 18 years old. The respondents are instructed to rate on a Likert scale the frequency with which the child demonstrates difficulty with the items described; it takes ∼ 10 to 15 minutes to complete and an additional 10 to 15 minutes to score. The self-report consists of 80 questions and is designed to be completed by the subject (aged 11 to 18 years old). 59

The BRIEF was translated and adapted to Portuguese in 2012 60 ; however, it has not been validated in Brazilian patients yet. In the USA, it has been recently used to assess executive function in a large sample of early-treated PKU patients (5 to 48 years old). Both young and older PKU patients scored in the “abnormally elevated” range of the working memory scale. In addition, executive function impairment appeared to affect more domains in the adult sample than in the child sample. Therefore, the authors concluded that working memory may be particularly affected in early-treated PKU patients. 17

#### Wechsler abbreviated scale of intelligence (WASI)

The Wechsler abbreviated scale of intelligence (WASI) was created to rapidly assess intelligence in patients from 6 to 89 years old. Its four subtests provide three composite scores: total IQ, verbal IQ, and the execution IQ. In addition, it allows the estimation of total IQ based on the application of two subtests, vocabulary and matrix reasoning. The application of the four-subtests form requires ∼ 30 minutes, while the two-subtests form requires an average of 15 minutes. The WASI was developed for people between the ages of 6 and 89 years old. 61 The internal validity and conceptual proposal of the Brazilian Portuguese version of the instrument were published in 2014. 62

One study showed that early-treated adult PKU patients have significant impairments in tasks involving complex executive functions, in particular functions involving planning/cognitive flexibility, monitoring and verbal reasoning assessed by vocabulary and similarities tests from the WASI. 18 However, in another study, no significant differences were found between the PKU group and the normative sample regarding the WASI Full Scale IQ or on the Matrix Reasoning or Vocabulary subtests. 63

#### SON-R 2½-7[a]

The SON-R 2½-7[a] is the shortened version of the SON-R 2½-7, a nonverbal intelligence test for children aged 2½ to 7 years old. This instrument has been standardized and validated in several countries in Europe and consists of six subtests: categories, analogies, situations, stories, mosaics, and patterns. The shortened version of the test comprises the subtests categories, situations, mosaics, and patterns, and was validated for use in Brazil in 2008 and was approved by the Federal Council of Psychology in 2012 as a psychological test fit for professional use. 64

Its purpose is the general assessment of developmental and cognitive abilities through four subtests that evaluate spatial, visuomotor, abstract, and concrete reasoning skills. It can be applied to both typical developing children and children with various types of disabilities; it is suitable for children with special language, speech, or communication needs, such as deafness, autism, and developmental disorders. 65 However, to date, there have been no studies using SON-R as a tool to assess these neuropsychological outcomes in PKU patients.

#### MTA-SNAP-IV or ASRS

The authors recommended two tools, The Multimodal Treatment Study for Attention-Deficit/Hyperactivity Disorder Swanson, Nolan, and Pelham, Version IV (MTA-SNAP-IV) and the Adult ADHD Self-Report Scale (ASRS), to support the diagnosis of ADHD associated with the clinical evaluation of the patient. These questionnaires can be used to assess ADHD symptoms in patients of different ages: the MTA-SNAP-IV is designed for use with children and adolescents, while the ASRA is for use with adults.

The SNAP-IV questionnaire was developed to evaluate symptoms of ADHD in children and adolescents. It can be completed by parents or teachers and consists of the symptoms listed in the Diagnostic and Statistical Manual of Mental Disorders (DSM-IV) for ADHD (criterion A) and oppositional-defiant disorder (ODD). MTA-SNAP-IV was the version used in the Multimodality Treatment Study, 66 which includes the 26 items corresponding to the DSM-IV Criterion A symptoms for ADHD and ODD symptoms, excluding other items present in previous versions. Each item evaluates the frequency of symptoms on a four-item Likert scale, ranging from 0 to 3. The MTA-SNAP-IV is sensitive to the effects of different treatments and has been translated into different languages, including Spanish, German, French, and Italian. It was translated into Brazilian Portuguese in 2006. 67

The ASRS is a self-administered adult ADHD symptom assessment. It was derived from the DSM-IV criteria for ADHD and comprises an 18-item instrument similar to the standard ADHD RS, with differences in language and scoring the frequency of symptoms (0–4) (“never,” “rarely,” “sometimes,” “often,” “very often”). The ASRS matches item for item domains of DSM-IV ADHD symptoms. 68 The original instrument in English has been adapted into a version to be used in Brazil. 69

MTA-SNAP-IV has recently been used to assess the prevalence of ADHD in patients with PKU in Brazil. 19 A cross-sectional study with a prospective neurological evaluation and retrospective collection of clinical information about ADHD in Brazilian patients with PKU showed that 16% of the patients had ADHD, in contrast to the expected 6% for the general population. 19

The influence of elevated Phe levels on ADHD symptoms has also been shown in the PKU ASCEND study. 70 The authors demonstrated that the use of sapropterin resulted in a significant improvement in the inattentive symptoms of ADHD in the first 4 weeks of treatment, suggesting that the reduction of Phe levels potentially reversed ADHD symptoms. In addition, a recent study showed that inattention symptoms improved among patients with PKU whose Phe levels decreased after treatment with pegvaliase, particularly those with high baseline inattention symptoms scores. 71

Although different tools were used to assess ADHD symptoms, these results corroborate the importance of ADHD evaluation in patients with PKU, suggesting that the reduction of symptoms could even be used as a therapeutic goal.

In addition to these tools recommended here, there are complementary tests that can provide additional data to support ADHD diagnosis. Therefore, whenever possible, we recommend the use of the Working Memory Index (IMO) (Digits, and Sequence of Numbers and Letters) of the Wechsler Intelligence Scale for Children (WISC- IV) and the Wechsler Intelligence Scale for Adults (Digits, Arithmetic and Sequence of Numbers and Letters) (WAIS-III). 72 The Wechsler Intelligence Scales for Children and the Wechsler Intelligence Scale for Adults are specific instruments that can be applied by a neuropsychologist to evaluate executive function, and some subtests of these tools such as the IMO have been shown to be capable of providing a great amount of information that can help in the diagnosis of ADHD and in the development of treatment plans.

## DISCUSSION

The authors herein describe and recommend tools that have been validated for use in Brazil for the neuropsychological assessment of PKU patients in daily clinical practice. It is a consensus among the experts that these evaluations should be applied to all patients with PKU (treated and non-treated). These tests should provide indexes of quality of life (Peds-QL, SF-36 or WHOQOL-bref), executive function (BRIEF or Bayley-III), IQ (SONR 2½-7[a] or WASI) and ADHD (MTA-SNAP-IV and ASRS).

The WASI scale takes ∼ 15 minutes (two subtests) to 30 minutes (four subtests), and the SON-R takes ∼ 20 to 30 minutes. The other scales vary from 5 to 15 minutes each. The recommendation is that the choice of tools should consider the setting of each center/service, as well as the age of the patients and, if possible, the tools should applied on the same day. However, it is important that the assessment does not exceed a total time of 1 hour and 15 minutes, so that the patient should not get tired, and to avoid possible biases.

It is the overall opinion that “the earlier the evaluation, the better”; since there are tests for early evaluations, such as the Bayley-III, which can be applied as soon as possible. The data from these tools will allow targeted and more effective interventions, which could prevent the long-lasting negative impacts of PKU. In addition, these neuropsychological and quality of life assessments will provide a more accurate understanding of the PKU burden on patients of different ages and with different treatment support management.

In Brazil, there is a significant number of patients with difficulties regarding treatment adherence due to financial problems, poor understanding of the benefits of the treatments, cognitive difficulties, and family conditions, among others. Moreover, the Brazilian Unified Health System (SUS, in the Portuguese acronym) sometimes is not able to ensure a regular supply of PKU metabolic formula to patients, which can have a negative impact on treatment adherence. 28 37 73

Indeed, it has already been shown that children in Brazil with PKU have lower performance regarding executive functions 74 and intellectual capacity compared with typically developing children, 75 which is probably due to unsatisfactory adherence to diet treatment. 37 The use of the assessments recommended in the present article may provide an integrated view of Brazilian patients, enabling a greater understanding of the obstacles involved in treatment adherence.

Moreover, these tools can assist in tackling the compromised neuropsychological functions present in individuals with PKU by enabling a better understanding of the effectiveness of different treatments. It may also allow a better understanding of the relationship between PKU and the quality of life of patients and their families with the condition.

As described above, the authors recommended only instruments that had already been validated for use in Brazil. However, there are two important PKU specific assessment tools that should be discussed here – PKU-QOL (PKU-Quality of Life) and PKU-SSIS (Symptom Severity and Impacts Scale) that have not yet been validated for use in Brazil.

The PKU-QOL was the first self-administered instrument developed for patients with PKU and their caregivers, and assesses three domains related to the disease and the impact of treatment. 29 It has been validated in eight countries (France, Germany, Italy, The Netherlands, Spain, Turkey, the United Kingdom, and the United States of America, but it has only been linguistically validated in Brazil). 32 The PKU-QOL questionnaire was well accepted by the participants of the linguistic validation study, which confirmed the relevancy of the original questions to Brazilian patients and their caregivers. 32

The PKU-SSIS is a patient-reported outcomes instrument designed to evaluate symptom severity and the impact of PKU on health-related quality of life, with a particular focus on neuropsychological symptoms and associated impacts. It includes 22 items, covering 3 symptom domains (emotional, mood, and psychological; neurocognitive, executive, and intellectual function; and physical health), and four impact domains (social relations, level of independence, general well-being, and selfcare). 76

PKU-SSIS is a recently published tool that has only been validated for early-treated adult PKU patients 76 ; however, as it combines the evaluation of neuropsychological and dietary factors to widely evaluate the impact of PKU on quality of life, it is expected that it will address an important gap in treatment monitoring.

It is essential to emphasize that these disease-specific assessments would provide even more detailed information about the disease and its treatment aspects, and they are already available for PKU patients in other countries; therefore, there should be a concerted effort to validate these tools for use in Brazil as soon as possible.

In conclusion, the instruments recommended in the present article that have already been validated for use in Brazil may be easily incorporated into daily clinical practice and help to improve the quality of the multidisciplinary care of patients with PKU. The experts' recommendations comprise nine tools ( Table 1 ) which assess quality of life (Peds-QL, SF-36 or WHOQOL-bref), executive function (BRIEF or Bayley-III), IQ (SONR 2½-7[a] or WASI) and ADHD (MTA-SNAP-IV and ASRS).
